# On a New Method of Treating the Effusion Which Collects under the Scull after Fractures of the Head

**Published:** 1802-03

**Authors:** J. Deveze

**Affiliations:** Officer of Health, of the first class, in the French Armies


### *On a new Method of treating the Effufion which collects under the Scull after Fractures of the Head. By* J. Deveze, *Officer of Health, of the firft clafs, in the French Armies.*

Of the different cafes which require the operation of the trepan, I fhall only confider the effufion between the dura mater and the fcull, occafioned by blows and fractures.

Mr. Petit, a celebrated furgeon of Paris, has contributed greatly to the improvement of this art, by pointing out the particular fymptoms which diftinguifh effufions under the fcull from concuffion of the brain. Thefe different accidents equally refult from falls or blows received on the head; and previous to this diftinction it was eafy to confound them, a miftake highly prejudicial to the patient who is affected with concuffion only, as it requires a different treatment from effufion, and is not relieved by the trepan.

When there is a collection of blood from a blow or fracture of the fcull, all authors advife the trepan, in order to difcharge the collected fluid; but the difficulty of afcertaining the part where it has accumulated, often makes frequent repetitions of the operation neceffary before it is difcovered. Mr. Marefchal, firft furgeon to Louis XIV. gives us an example of this; he trepanned trepanned a young lady twelve times before he found the effufion occafioned by a fracture of the parietal and temporal bones on the fame fide. ' This cafe, and many others of a fimilar kind too numerous to relate, evidently fhow how important it is to render an operation more eafy, which is often repeated without real neceffity, is painful to the operator, and fometimes fatal to the patient.

I do not flatter myfelf with having obtained this object; but I think it a duty to communicate to the public fome ideas which have occurred to me, and by which I have been fo happy as to fucceed, in a cafe I had an opportunity of attending in the French hofpital eftablifhed at Philadelphia.

In cafes of accumulated blood between the fcull and dura mater, the adhefion which unites them is deftroyed in the place occupied by the fluid, the collected matter is circumfcribed in a larger or fmaller fpace, it expands the dura mater, and forming a tumor that oppreffes the brain, produces the effects which require the operation of the trepan.

In this cafe a fingle opening made in the fcull on one of the points of effufion, is fufficient to give vent to the fluid, becaufe the blood, preffed on all fides by the action of the brain, quits the place it had collected in, and flows towards a part that offers a paffage. It happens in this cafe, as it does in that where the accumulation exifts between the dura and pia mater, that the blood, equally preffed by the brain, runs between thofe two membranes, flows towards the opening by the trepan, and preffes the dura mater outward, which indicates to the operator that this membrane requires incifion, in order to give paffage to the collected fluid.

It is only in the firft cafe that the adhefion of the dura mater to the cranium, by retaining the fluid, requires a repetition of the opening, fhould the operator not meet at firft with the precife fpot where the fluid is collected.

To avoid thefe inconveniencies, I propofe in fuch a cafe to deftroy the adhefion which unites the dura mater to the fcull, and eftablifh a communication between the collected blood and the opening already made by the trepan; by this means a repetition of trepanning would be avoided, and the operation becoming more eafy might fave the life of the patient in any cafe not neceffarily mortal, which is particularly interefting when the effufion is fituated at the bottom of the fcull.

If inftead of trepanning twelve times, Mr. Marefchal had feparated the dura mater from the cranium, following the direction of the fracture, he would have certainly reached the effufion, and the blood would have been evacuated by the firft opening, although it muft have rifen againft it own weight; this this will be eafily underftood by phyfiologifts, who advert to the force of preffure the brain exercifes on every part of the fcull, and compare it with the refiftance the collected blood may oppofe by its fpecific weight.

The danger arifing from a feparation of the dura mater, may perhaps be confidered as forbidding the method I recommend; but experience fhews this feparation is not dangerous, fince, as I have already faid, blood cannot collect between thefe two parts, without feparation, and yet they return to their natural ftate, when the fluid is evacuated by the trepan, even where the feparated parts have long remained divided from each other by the interpofition of the fluid.

Of the cafes which fupport my opinion, it will be fufficient to mention the following.

A young perfon, after the fcarlet fever, had a violent pain which fixed itfelf at the upper part of the head; every thing art could indicate was tried to effect a cure, bleeding, bathing, cathartics, internal remedies, topicals of every kind, and bliffters on the affected part, all had failed; when I was confulted, I advifed the moxa, which was applied to the difeafed part, and though a plentiful fuppuration followed, the pain feemed to increafe, and for fix months continued to augment. When I was again requefted to give my advice, I prefcribed the trepan, which operation was immediately performed, in the centre of the painful part; the opening made in the fcull by this means gave vent to a quantity of pus of a greenifh white colour; the pain ceafed entirely, the patient was foon cured; and fince has enjoyed a perfect ftate of health.

The preceding obfervation clearly fhews the dura mater had been long feparated from the fcull by the matter, and proves that the feparation of this membrane is not dangerous.

It will be faid, perhaps, that this feparation did not produce any bad effect, becaufe it took place gradually; my anfwer is, an effufion occafioned by violent blows is fuddenly formed, it forces the dura mater from the cranium with violence, and feparates it fometimes to a great extent. It may be again objected that nature, though acting haftily, manages in a manner art cannot imitate in feparating the dura mater from the fcull. I will oppofe this objection by experience, and not by argument.

The 29th of March, 1795, there was brought to the French hofpital eftablifhed in this city, a man about thirty-eight years of age, of a middle fize and very robuft conftitution: he was comatofe, his face inflated and difcoloured with ecchymofis, his body covered with bruifes, and many wounds made with pointed inftruments. Thofe who brought him, told me he had been ftruck

ſtruck with an iron bar, which fractured his ſcull; and had been trepanned on the ſpot.

After uncovering the head, it was waſhed and ſhaved; and I found the trepan had been applied on the upper part of the right parietal bone, about an inch from the coronal ſuture.

I took away, with the lenticular knife, pieces of the internal plate which wounded the dura mater, and enlarged the wounds in the direction of the fracture on which the trepan had been applied; it proceeded from the ſagittal ſuture, and deſcended almoſt in a right line into the temporal region, at the upper part of which I bounded my inciſion, although the fracture extended lower; I obſerved another fracture in the upper and lower part of the ſame parietal, which had ſeparated a piece of the bone about three inches long, and two wide; this piece was neither indented nor diſplaced, was behind and a little above the part trepanned: blood iſſued from the ſuperior and poſterior fractures.

The next morning the patient was in the ſame ſtate, inſenſibly voided his urine, and could not ſwallow. The dreſſing was removed, much blood came from the opening by the trepan, and from the fractures.

When I viſited him in the evening he was comatoſe; but little blood came from the wound, nor did the preſſure I made on the dura mater produce more. I introduced a blunt flexible probe under the ſcull, in the direction of the fracture, from whence the blood proceeded in the laſt dreſſing, and endeavoured to do the ſame by the fracture which deſcended to the temporal region; at the diſtance of about a quarter of an inch I was ſtopped by a ſudden reſiſtance, and it was at that moment reflection ſuggeſted the method I immediately put in practice.

I preſumed the comatoſe drowſineſs which continued, was occaſioned by collected blood, and that it exiſted under one of the points of the fracture in the temporal region, becauſe thoſe effuſions which had been formed under the other fractures, were evacuated in the preceding dreſſing.

Had I followed the uſual method, it is poſſible I might have made many openings before I had ſucceeded, or have failed finding it; conſequently, after the reflection which ſuggeſted that the adheſion between the ſcull and the dura mater might be ſeparated without inconvenience, I determined to ſeparate the membrane by following the direction of the fracture; and proceeded to this operation with a ſilver ſpatula very flexible, the extremities of which were rounded: I took the precaution to preſs it towards the bone, and to bend my inſtrument by degrees as I entered, to make it take the form of the part upon which which I acted, and often drew it back, to meafure on the outfide the way it had made. At length, after having entered half an inch below the temporal fcaly future, the refiftance fuddenly ceafed, and my inftrument entered a hollow part, at the fame moment the blood flowed in great abundance; when it ceafed I drew out the fpatula, which was followed by a fmall quantity. The patient then began to move ftrongly, tried to rife and talk, without knowing what he faid.

The next morning I found him tied in his bed; this method was neceffary, becaufe he endeavoured to rife, as he faid, to go and fight. More blood came away at the dreffing. In the afternoon I found him better; he drank plentifully, and anfwered my queftions: the next morning, being the fourth after the accident, he had perfectly recovered his fenfes, and from that time continued to mend. As his head had been much wounded, many abfceffes were formed on the exterior; the laft was on the piece of the parietal bone already fpoken of, and as it had no connection, and was vacilating, I eafily took it away, and the dura mater recovered and followed the motion of the brain: the wound had fuppurated and the cicatrix was much advanced, when the patient went out the 28th of December.

During the cure, the patient felt no pain in that part of the head where I had feparated the dura mater; the cure of this trepan was neither longer nor more difficult than ufual, if we except the complication from the gatherings, which is foreign to the fubject.

I cited Mr. Marefchal's obfervation, becaufe the cafe is fimilar to that which makes the fubject of this effay. Mr. Marefchal's patient had a fracture which croffed the parietal and temporal bones: mine had fractures in the fame bones, and fame places; there was alfo another, and fome very ferious bruifes, which made the difeafe complicated. Mr. Marefchal trepanned his patient twelve times; I cured mine with one operation, and by a method which, to the beft of my knowledge, had never before been tried.

